# Association of serum N-terminal pro-brain natriuretic peptide levels with survival and renal outcomes among elderly patients with acute kidney injury in chronic heart failure

**DOI:** 10.3389/fcvm.2023.1104787

**Published:** 2023-02-03

**Authors:** Jiebin Hou, Xin Zhang, Zhen Wu, Yang Liu, Yabin Zhang, Jiahui Zhao, Xiaohua Wang, Hongyu Chen, Guang Yang, Qiang Ma, Qingli Cheng, Qiangguo Ao

**Affiliations:** ^1^Department of Nephrology, The Second Medical Center & National Clinical Research Center for Geriatric Diseases, Chinese People’s Liberation Army (PLA) General Hospital, Beijing, China; ^2^Institute of Geriatrics, The Second Medical Center, Chinese People’s Liberation Army (PLA) General Hospital, Beijing, China

**Keywords:** chronic heart failure, natriuretic peptides, acute kidney injury, elderly, prognosis

## Abstract

**Background:**

Elderly patients exhibit a higher incidence of chronic heart failure (CHF). Patients with CHF can develop acute kidney injury (AKI) during follow-up, which can result in poor prognosis. This relationship between kidney dysfunction and levels of N-terminal pro-brain natriuretic peptides (NT-proBNP), with regard to prognosis, is complicated and has rarely been analyzed in elderly patients with CHF.

**Method:**

We conducted a retrospective cohort study involving patients with a CHF history aged ≥ 65 years, who experienced an episode of AKI. Kaplan–Meier curves and Cox or logistic proportional hazards regression models were used to evaluate the association between serum NT-proBNP concentrations and mortality or renal recovery by day 90.

**Results:**

A total of 1,160 eligible patients with AKI were available for the study. Of this sample, 41.5% of patients died within 90 days of the onset of AKI. Patients with a decreased change in NT-proBNP accompanying the episode of AKI had a lower risk (adjusted OR = 0.56, 95% CI = 0.34−0.91) of more severe AKI (stage 2 and 3 vs. stage 1). The more severe AKI were associated with higher mortality and non-recovery of renal function in elderly patients with CHF, independent of NT-proBNP levels. Elevated levels of baseline lnNT-proBNP (adjusted HR = 1.27, 95% CI = 1.17−1.38) predicted mortality in elderly patients with CHF within 90 days of AKI onset. Patients with a decrease in NT-proBNP accompanying AKI had a lower risk of mortality (adjusted HR = 0.62, 95% CI = 0.48−0.79). However, a decrease in NT-proBNP is a risk factor (adjusted OR = 1.59, 95% CI = 1.02−2.48) for the non-recovery of renal function following AKI–especially in elderly survivors with low baseline NT-proBNP levels.

**Conclusion:**

A decreased change in NT-proBNP maybe protective for elderly patients with CHF by improving survival outcomes and preventing severe AKI. However, an excessive decrease in NT-proBNP is a risk factor for the non-recovery of renal function following AKI. Avoiding excessive changes in NT-proBNP may be protective for survival and renal injury prognosis.

## 1. Introduction

The prevalence of chronic heart failure (CHF) is high and increasing in aging populations worldwide. The higher incidence of CHF in elderly persons can be attributed to aging and the high frequency of comorbidities, particularly coronary heart disease and hypertension. CHF with preserved ejection fraction is particularly prevalent in geriatric patients. The aging-related changes in the diastolic properties of the myocardium predispose older adults to develop CHF with preserved ejection fraction and atrial fibrillation (1). Elderly patients with heart failure (HF) experience high mortality rates. Compared to younger patients, elderly patients with HF are characterized by more severe clinical conditions, an increased proportion of non-cardiovascular death, and poorer prognoses (2). Considering the high incidence of CHF and poor CHF prognoses in the elderly, more CHF-related studies focusing on this vulnerable patient group are necessary.

B-type natriuretic peptide (BNP) and the precursor of BNP, N-terminal pro-brain natriuretic peptide (NT-proBNP), are produced mainly by cardiomyocytes in response to stretch from volume overload and myocardial ischemia. Circulating natriuretic peptides are widely used biomarkers that are indicative of HF and volume overload. In elderly patients with heart disease, elevated NT-proBNP levels are associated with a greater risk of cardiovascular mortality (3). In elderly patients with CHF, diuretics are frequently utilized as decongestion treatment, which accompanies a decrease in BNP level (4).

Patients with CHF are often characterized by impaired renal function, also referred to as cardiorenal syndrome (CRS). Cardiorenal syndrome is prevalent in the elderly, and a dynamic interplay between the heart and kidneys exists (5). Patients with CHF can develop chronic renal dysfunction or acute kidney injury (AKI) during follow-up. In elderly patients with CHF, pre-renal AKI is frequent due to decreased renal perfusion caused by HF or depletion of effective circulating volume. Elderly patients are more prone to the development of volume depletion because of their restricted ability to access fluids and the frequent use of diuretics (6). AKI is associated with an increased mortality risk that is proportional to the severity of the AKI (7). Furthermore, many patients cannot recover from AKI and thus, progress to more severe stages of chronic kidney disease (CKD) (8). Renal injury is associated with a higher risk of adverse outcomes and is also a predictor of all-cause mortality and rehospitalization in HF (9). However, recent findings indicate that improving renal function is paradoxically associated with worse outcomes in acute HF, but outcomes may differ based on the response to decongestion (decreased change in NT-proBNP) (4).

Since most of the randomized clinical trials for CHF and AKI were designed to exclude elderly patients, only limited data on elderly patients with cardiorenal syndrome are available. In the current analysis, we conducted a retrospective observational study to investigate the relationships between change in NT-proBNP, renal function and adverse outcomes in elderly patients with AKI in CHF.

## 2. Materials and methods

### 2.1. Study design and cohort formation

We conducted a retrospective cohort study at the Second Medical Center (Geriatric Department) of the Chinese PLA General Hospital to assess AKI outcomes in older patients with CHF. Patients 65 years of age or older, with a history of CHF with preserved and mildly reduced ejection fraction [left ventricular ejection fraction (LVEF) > 40%], were enrolled if they experienced an episode of AKI between 1 January 2008 and 31 December 2018. Patients with end-stage renal disease (eGFR < 15 mL/min/1.73 m^2^) or incomplete medical histories were excluded from the study. Patients were also excluded if they lacked serum creatinine (SCr) or NT-proBNP data, either at baseline or at AKI onset. We followed all patients for 90 days after the initial AKI episode, to determine clinical outcomes relating to mortality and renal function.

This study was conducted in accordance with the Declaration of Helsinki and was approved by the ethics committee of the Chinese People’s Liberation Army General Hospital (S2022-342-01), and the requirement for informed consent was waived.

### 2.2. Clinical definitions and outcomes

The ICD10 codes I50.22 and I50.32 were used to retrieve data on patients with CHF history. The diagnosis of CHF was based on clinical findings, symptoms, abnormal electrocardiogram, elevation of brain natriuretic peptide level (NT-proBNP > 125 pg/mL), and structural cardiac abnormalities identified on echocardiography according to 2021 ESC guidelines (10). To select patients who experienced an episode of AKI, AKI was defined and classified based on the KDIGO (Kidney Disease Improving Global Outcomes) criteria, using SCr and urine output criteria (11). AKI is defined as any of the following: increase in SCr by ≥0.3 mg/dl (≥26.5 μmol/l) within 48 h; or increase in SCr to ≥1.5 times baseline, which is known or presumed to have occurred within the prior 7 days; or urine volume < 0.5 ml/kg/h for 6 h. AKI was classified into three stages for severity. Baseline SCr values were obtained in a stable state within 3 months of AKI onset. The estimated glomerular filtration rate (eGFR) was calculated using the Chronic Kidney Disease Epidemiology Collaboration (CKD-EPI) 2009 equation. AKI at stage 2 or 3 was defined as more severe AKI. All patients were followed up for 90 days after the initial AKI diagnosis, to assess primary outcomes and all-cause 90-day mortality.

The secondary outcome of the study was non-recovery from AKI, defined as the last available SCr measurement (during the follow-up of 90 days) where SCr remained at more than 150% of the baseline value (thus still meeting the criteria for AKI).

### 2.3. Data collection

All data were obtained from hospital electronic health records. Demographic characteristics (age and sex), medical history [including LVEF value, the use of medication (ACEI or ARB, beta-blockers, aldosterone antagonist, loop diuretics), hypertension, diabetes mellitus, hyperlipidemia, coronary heart disease, prior myocardial infarction, atrial fibrillation, chronic obstructive pulmonary disease (COPD), and malignant tumors], accompanying conditions (proteinuria, extremity edema, infection, and blood pressure), and laboratory test results [including plasma SCr, NT-proBNP, hemoglobin, cardiac troponin I (cTnI), glucose, potassium, calcium, and phosphorus] were recorded. SCr levels were routinely measured in our clinical laboratory, using the Cobas c 501 analyzer (Roche Diagnostics). The serum NT-proBNP concentration was measured using Dimension EXL with an LM Integrated Chemistry System (Siemens). Within 3 months before AKI onset, the NT-proBNP level at baseline (NT-proBNP at baseline) was measured together with the baseline SCr value in a stable state. The NT-proBNP at AKI diagnosis (NT-proBNP at AKI) was measured together with the increased SCr value, which contributed to the diagnosis of AKI. The change in NT-proBNP (continuous) accompanying the episode of AKI was expressed as the ratio of NT-proBNP _at AKI_ to NT-proBNP _at baseline_. The change in NT-proBNP was also analyzed as a binary variable. A decreased change in NT-proBNP was defined by the ratio of NT-proBNP _at AKI_ to NT-proBNP _at baseline_ < 1.

### 2.4. Statistical analysis

Continuous variables were presented as mean ± standard deviation for normally distributed data or median [25−75% interquartile ranges (IQR)] for non-normal distributions. Discrete variables were presented as percentages. Patient characteristics were compared by the Student’s t-test, the Mann–Whitney *U* test (continuous variables), or Pearson’s χ^2^ test (categorical variables). NT-proBNP concentrations were log-transformed to reduce skew, modeled as continuous variables, and divided into quartiles. Kaplan–Meier, log rank, and univariate and multivariate Cox regression analyses were used for the outcomes of 90-day mortality. Univariate and multivariate logistic regression models were used to evaluate the associations between NT-proBNP concentrations and the severity of AKI and the 90-day renal recovery. Multivariable models were constructed including NT-proBNP levels, severe AKI and confounding factors that were significantly associated with outcomes in the univariate analyses (*P* < 0.05). Interaction testing was performed between the direction of change in NT-proBNP, before and after AKI, and the quartiles of baseline NT-proBNP levels, with renal outcomes. The association between the change in NT-proBNP levels and non-recovery from AKI was further examined within different quartiles of baseline NT-proBNP levels. *P*-values of < 0.05 were considered statistically significant for all analyses, including interaction terms.

## 3. Results

### 3.1. Clinical characteristics and survival outcomes of patients

A total of 1,160 eligible patients with CHF and AKI were enrolled and assessed in this study. Clinical characteristics and survival outcomes of the enrolled geriatric patients are shown in Table 1. A total of 482 patients (41.5%) died within 90 days of the onset of AKI. The age of non-survivors was higher than that of the survivors. Substantially elevated NT-proBNP levels (at baseline and at AKI diagnosis) and greater changes in NT-proBNP levels (ratio of NT-proBNP _at AKI_/NT-proBNP _at baseline_) were found in the non-survivors. Stage 1 AKI occurred in 746 patients (64.3%), stage 2 in 245 patients (21.1%), and stage 3 in 169 patients (14.6%). In the non-surviving group, the percentage of patients with more severe AKI was higher than that in the surviving group (stage 3, 28.4 vs. 4.7% and stage 2, 25.7 vs. 17.8%, respectively). Moreover, non-survivors experienced a higher incidence of comorbidities including prior myocardial infarction, atrial fibrillation, COPD, and malignant tumors, as well as abnormal conditions including lower LVEF, proteinuria, extremity edema, infection, higher blood glucose, electrolyte disturbance, lower blood pressure, lower hemoglobin and higher cTnI. Additionally, survivors were more often treated with angiotensin-converting enzyme inhibitors (ACEIs) or angiotensin receptor blockers (ARBs), and non-survivors were more often treated with loop diuretics.

**TABLE 1:** Patient characteristics between elderly survivors and non-survivors.

	Non-survivors (*n* = 482)	Survivors (*n* = 678)	*P*-value
Age (years), median (IQR)	91 (87,95)	90 (87,94)	0.044
Male, *n* (%)	432 (89.6)	595 (87.8)	0.350
Baseline eGFR, (mL/min/1.73 m^2^), median (IQR)	67.2 (44.5,82.6)	70.1 (48.3,83.0)	0.196
NT-proBNP _at baseline_ (pg/mL), median (IQR)	2122 (790,5830)	907 (361,2512)	<0.001
NT-proBNP _at AKI_ (pg/mL), median (IQR)	4460 (1731,12173)	1570 (526,4488)	<0.001
Ratio of NT-proBNP _at AKI_/ NT-proBNP _at baseline_, median (IQR)	1.80 (1.02,3.75)	1.23 (0.88,2.56)	<0.001
**AKI stages**			<0.001
Stage 1 AKI, *n* (%)	221 (45.9)	525 (77.4)	-
Stage 2 AKI, *n* (%)	124 (25.7)	121 (17.8)	-
Stage 3 AKI, *n* (%)	137 (28.4)	32 (4.7)	-
**LVEF, *n* (%)**			<0.001
40% < LVEF < 50%	163 (33.8%)	90 (13.3%)	-
EF ≥ 50%	319 (66.2%)	588 (86.7%)	-
**Medications, *n* (%)**			
ACEI or ARB	67 (13.9%)	231 (34.1%)	<0.001
Beta-blockers	72 (14.9%)	122 (18.0%)	0.169
Aldosterone antagonist	125 (25.9%)	195 (28.8%)	0.288
Loop diuretics	195 (40.5%)	161 (23.7%)	<0.001
**Comorbidities, *n* (%)**			
Hypertension	350 (72.6)	544 (80.2)	0.003
Diabetes mellitus	244 (50.6)	379 (55.9)	0.083
Hyperlipidemia	190 (39.4)	325 (47.9)	0.004
Coronary heart disease	388 (80.5)	581 (85.7)	0.020
Prior myocardial infarction	165 (34.2)	165 (24.3)	<0.001
Atrial fibrillation	159 (33.0)	179 (26.4)	0.015
COPD	192 (39.8)	231 (34.1)	0.044
Malignant tumor	229 (52.4)	208 (47.6)	<0.001
**Information at AKI**			
Proteinuria, *n* (%)	288 (59.8)	357 (52.7)	0.017
Extremity edema, *n* (%)	214 (44.4)	204 (30.1)	<0.001
Infection^a^	275 (57.1)	235 (34.7)	<0.001
Fasting glucose (mmol/L), median (IQR)	8.54 (6.37,11.61)	6.9 (5.56,9.40)	<0.001
Potassium (mmol/L), median (IQR)	4.40 (3.91,4.90)	4.27 (3.90,4.70)	0.002
Calcium (mmol/L), median (IQR)	2.20 (2.04,2.37)	2.24 (2.13,2.37)	<0.001
Phosphorus (mmol/L), median (IQR)	1.20 (0.90,1.51)	1.10 (0.90,1.30)	<0.001
Systolic blood pressure (mmHg), median (IQR)	111 (100,124)	123 (109,136)	<0.001
Diastolic blood pressure (mmHg), median (IQR)	57 (50,66)	62 (55,70)	<0.001
Hemoglobin (g/dL), median (IQR)	95 (82,110)	109 (95,122)	<0.001
cTnI (μg/L), median (IQR)	0.108 (0.04,0.44)	0.04 (0.01,0.11)	<0.001

^a^Infection including: respiratory tract infection, gastrointestinal infection, urinary tract infection, skin and soft tissue infection, and fever of unknown origin.

### 3.2. AKI stages and NT-proBNP

As shown in Supplementary Table 1, a higher baseline NT-proBNP level is a protective factor [adjusted odds ratio (OR) = 0.78 95% CI = 0.68−0.89] against more severe stage AKI. Patients with the highest quartile of baseline NT-proBNP (Q4: NT-proBNP > 3,729 vs. Q1: NT-proBNP ≤ 480) had a lower incidence of more severe AKI. However, patients with greater changes in NT-proBNP represented as the ratio of NT-proBNP at AKI/NT-proBNP at baseline, had a significantly higher incidence of more severe AKI. Patients with a decreased change in NT-proBNP accompanying the episode of AKI had a lower risk (adjusted OR = 0.56, 95% CI = 0.34−0.91) of severe AKI.

Severe AKI may result from several associated risk factors. Supplementary Table 2 shows that infection, higher fasting blood glucose, elevated phosphorus, lower hemoglobin, use of loop diuretics and non-use of ACEI/ARB were risk factors for more severe AKI. A lower level of baseline NT-proBNP and an increased change in NT-proBNP at AKI were associated with a severe AKI episode.

### 3.3. Survival analysis and hazard ratio for 90-day mortality based on NT-proBNP or AKI stages

**FIGURE 1:**
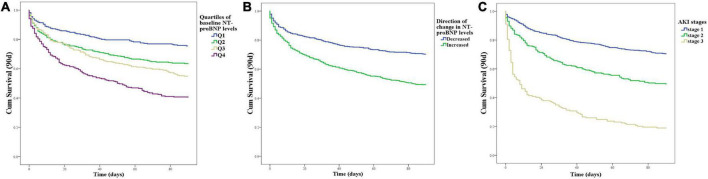
Patient survival on day 90 with respect to the NT-proBNP levels or AKI stages. **(A)** Kaplan–Meier curves for 90-day mortality with respect to the four quartiles of baseline NT-proBNP levels at baseline. **(B)** Kaplan–Meier curves for 90-day mortality with respect to the direction of change in NT-proBNP levels accompanied by an episode of AKI. **(C)** Kaplan–Meier curves for 90-day mortality with respect to the AKI stages.

Kaplan–Meier curves for 90-day mortality showed that patients with higher NT-proBNP levels at baseline (log rank, *P* < 0.001; Figure 1A) had higher mortality concerning the four quartiles of NT-proBNP levels. Patients with a decreased change in NT-proBNP had significantly higher survival rates (Figure 1B). With regard to AKI stages, the 90-day survival rate ranged from 70.4% in those with stage 1 to 49.4% and 18.9% in those with stage 2 and 3 AKI, respectively. Kaplan–Meier curves (Figure 1C) showed significantly increased mortality in stage 2 (log rank *P* < 0.001) and stage 3 (log rank *P* < 0.001) compared to stage 1.

The Cox proportional hazard analyses (Table 2) showed that baseline lnNT-proBNP was associated with mortality in the unadjusted and adjusted models [adjusted hazard ratio (HR) = 1.27, 95% CI = 1.27−1.38]. A significant association between the direction of change in NT-proBNP levels and mortality was found. Patients with a decrease in NT-proBNP at the time of AKI diagnosis had a lower risk of mortality (adjusted HR = 0.62, 95% CI = 0.48−0.79). However, the continuous change in NT-proBNP, expressed by the ratio of NT-proBNP at AKI to NT-proBNP at baseline, was not significantly associated with mortality. Compared with those with stage 1 AKI, patients with more severe AKI had higher unadjusted and adjusted HR (1.52, 95% CI = 1.20–1.93) for 90-day mortality. Factors considered for the adjustments in Cox proportional hazard analyses for mortality are listed in Supplementary Table 3. In multivariable model, Lower LVEF, non-use of ACEI/ARB, malignant tumors, infection, higher blood glucose, higher phosphorus lower hemoglobin level, higher cTnI and lower systolic pressure were significantly associated with 90-day mortality.

**TABLE 2:** Univariable and multivariable Cox regression analysis for 90-day mortality by AKI stages and NT-proBNP levels.

	Unadjusted HR (95% CI)	Unadjusted *P* value	Adjusted HR (95% CI) in model 1^a^	Adjusted *P* value^a^	HR (95% CI) in model 2^b^	Adjusted *P* value^b^
More severe AKI	2.02 (1.62, 2.52)	<0.001	2.12 (1.70, 2.66)	<0.001	1.52 (1.20−1.93)	0.001
ln NT-proBNP _atbaseline_	1.37 (1.28, 1.46)	<0.001	1.46 (1.36, 1.56)	<0.001	1.27 (1.17, 1.38)	<0.001
**Change in NT-proBNP**						
Ratio of NT-proBNP _atAKI_ to NT-proBNP _atbaseline_	1.008 (1.00,1.02)	0.075	1.01 (1.01,1.02)	0.001	1.01 (1.00,1.02)	0.007
Decreased change in NT-proBNP^c^	0.55 (0.44,0.70)	<0.001	0.49 (0.38,0.61)	<0.001	0.62 (0.48−0.79)	<0.001

^a^Mortality model 1: ln NT-proBNP at baseline, change in NT-proBNP, severe AKI.

^b^Mortality model 2: ln NT-proBNP at baseline, change in NT-proBNP, severe AKI, LVEF, Loop diuretics, ACEI/ARB, hypertension, hyperlipidemia, coronary heart disease, prior myocardial infarction, atrial fibrillation, COPD, malignant tumor, edema, infection, glucose, potassium, calcium, phosphorus, hemoglobin, cTnI, systolic blood pressure and diastolic blood pressure.

^c^Ratio of NT-proBNP _at AKI_/NT-proBNP _at baseline_ < 1 compared to ratio of NT-proBNP _at AKI_/NT-proBNP _at baseline_ ≥ 1.

### 3.4. Renal recovery and NT-proBNP

Renal outcomes were evaluated in 678 survivors on day 90 after the episode of AKI. The renal function of 122 (18%) survivors did not recover from AKI. Significant variables (baseline eGFR, loop diuretics, atrial fibrillation, malignant tumor, proteinuria, glucose, calcium, and hemoglobin) from univariate logistic regression analyses and retained adjustors (NT-proBNP levels and AKI stages) were analyzed in multivariate models (Supplementary Table 4) as covariates. As shown in Table 3, the baseline NT-proBNP level was not associated with non-recovery in the unadjusted and adjusted logistic regression models. However, the decreased change in NT-proBNP level was shown to be a risk factor (adjusted OR = 1.59, 95% CI = 1.02−2.48) in the non-recovery of renal function. Patients with more severe AKI were associated with the non-recovery of renal function.

**TABLE 3:** Univariable and multivariable logistic regression analysis for 90-day renal non-recovery by AKI stages and NT-proBNP levels.

	Unadjusted OR (95% CI)	Unadjusted *P* value	Adjusted^a^ OR (95% CI) in model 1^a^	Adjusted^a^ *P* value	Adjusted^b^ OR (95% CI) in model 2^b^	Adjusted^b^ *P* value
More severe AKI	1.88 (1.04, 3.43)	0.038	2.12 (1.15, 3.90)	0.016	2.11 (1.09, 4.10)	0.026
ln NT-proBNP _at baseline_	1.04 (0.89, 1.21)	0.610	1.03 (0.88, 1.20)	0.714	1.01 (0.84, 1.21)	0.921
**Change in NT-proBNP**						
Ratio of NT-proBNP _at AKI_ to NT-proBNP _at baseline_	0.90 (0.83, 0.98)	0.010	0.89 (0.81, 0.97)	0.006	0.90 (0.83, 0.98)	0.014
Decreased change in NT-proBNP^c^	1.55 (1.03, 2.33)	0.034	1.63 (1.07, 2.48)	0.022	1.59 (1.02, 2.48)	0.041

^a^Mortality model 1: lnNT-proBNP at baseline, change in NT-proBNP, severe AKI.

^b^Mortality model 2: lnNT-proBNP at baseline, change in NT-proBNP, severe AKI, baseline eGFR, loop diuretics, atrial fibrillation, malignant tumor, proteinuria, glucose, calcium and hemoglobin.

^c^Ratio of NT-proBNP _at AKI_/NT-proBNP _at baseline_ < 1 compared to ratio of NT-proBNP _at AKI_/NT-proBNP _at baseline_ ≥ 1.

Interaction testing between the baseline NT-proBNP and the ratio of NT-proBNP _at AKI_ to NT-proBNP _at baseline_ for non-recovery of renal function was not statistically significant. However, interaction testing was significant (adjusted *P* = 0.026) for the interaction between the direction of change in NT-proBNP and the quartiles of baseline NT-proBNP levels. In the lowest quartile of baseline NT-proBNP (Q1, NT-proBNP ≤ 480), patients with decongestion accompanied by an episode of AKI had significantly worse renal outcomes (Table 4). Meanwhile, among patients with higher NT-proBNP levels at baseline (from Q2 to Q4), a decreased change in NT-proBNP levels did not significantly affect renal outcomes.

**TABLE 4:** Odds ratios for 90-day renal non-recovery associated with decreased change in NT-proBNP within quartiles of baseline NT-proBNP levels.

	Unadjusted			Adjusted^a^		
	OR (95% CI)	*P* value	*P* value for interaction	OR (95% CI)	*P* value	*P* value for interaction
**NT-proBNP_at baseline_**						
Overall	1.55 (1.03−2.33)	0.034	0.002	1.66 (1.08, 2.56)	0.022	0.026
Q1 ≤ 480	4.72 (2.23, 9.99)	<0.001		3.94 (1.67, 9.28)	<0.002	
Q2 (480−1,295)	1.06 (0.46, 2.44)	0.889		1.07 (0.44, 2.58)	0.887	
Q3 (1,295−3,729)	1.21 (0.55, 2.67)	0.634		1.21 (0.53, 2.78)	0.657	
Q4 > 3729	0.64 (0.24, 1.74)	0.383		0.83 (0.29, 2.40)	0.725	

^a^With adjustment for more severe AKI, baseline eGFR, loop diuretics, proteinuria, and fasting glucose.

## 4. Discussion

Chronic heart failure is a common disease in the elderly, with the prognosis worsening with age (12). Chronic and acute renal insufficiency are highly prevalent in patients with CHF and associated with poor outcomes (13). According to previous studies (14, 15), the prevalence of heart failure with preserved ejection fraction (HFpEF) or mildly reduced ejection fraction (HFmrEF) increases sharply with age. Therefore, elderly patients with a history of HFmrEF or HFpEF (LVEF > 40) were included in this study.

Elderly patients are often frail and have multiple comorbidities (such as hypertension and coronary heart disease), which could contribute to the decline in heart and renal function, leading to the deterioration of systemic functions and worse outcomes (16). Elderly patients with HFmrEF or HFpEF had more comorbidities and died more often from non-cardiovascular causes, compared to those with heart failure with reduced ejection fraction (HFrEF) (15, 17). Infection, malignant tumors and lower systolic pressure were significantly associated with 90-day mortality in patients with AKI in CHF according to this study.

Elderly patients have the highest prevalence of HF, and a positive correlation between natriuretic peptides and age has been found (18). Natriuretic peptides are released from the heart, in response to wall stretch induced by volume or pressure overload. BNP and NT-proBNP correlate with markers of cardiac dysfunction and volume overload and are clinically used as valuable diagnostic and prognostic markers of HF (19). Due to the longer half-life of NT-proBNP compared to that of BNP (120 vs. 22 min), NT-proBNP is more stable in reflecting changes in hemodynamics. Natriuretic peptides are predictors of all-cause mortality in patients with HF, as previously reported (20). NT-proBNP-guided decongestive treatment has been suggested in patients with CHF (21). According to our study, both high levels of baseline NT-proBNP and an increased change in NT-proBNP at follow-up were associated with a higher risk of all-cause mortality. A decreased change in NT-proBNP, accompanying the episode of AKI was considered protective for survival in elderly patients with CHF.

Cardiorenal syndrome is a common clinical condition in the elderly. Cardiorenal syndrome occurs when dysfunction of either the heart or kidneys progresses to the other organ, leading to both cardiac and renal failure. Patients with CHF can develop AKI during follow-up (13). Pre-renal AKI is prevalent in patients with HF due to decreased renal perfusion from any cause, such as HF or depletion of effective circulating volume (6). Natriuretic peptides are important biomarkers of cardiorenal syndrome and play essential roles in its progression (22). Elevated NT-proBNP/BNP ratios were found to predict worsening renal function in patients with acute HF (23). Many mechanisms have been proposed to explain the decline in renal function in patients with HF, including low cardiac output and renal congestion due to volume overload (24). Elevated NT-proBNP was a significant independent predictor for the accelerated progression of renal dysfunction to end-stage kidney disease, after adjustment for other variables was made (25). In our study, an increased change in NT-proBNP, rather than a high baseline level of NT-proBNP, was found to be associated with more severe AKI. Thus, continuous monitoring of NT-proBNP is recommended in patients with CHF.

The role of kidney dysfunction in mortality has been frequently observed in patients with HF, especially acute decompensated HF. Improving renal function was previously reported to be paradoxically associated with worse outcomes in acute HF, but outcomes may differ based on the response to decongestion (decrease in NT-proBNP) (4). The relationship of kidney dysfunction with mortality in acute HF was not independent on the congestion status and differed by BNP trajectory. Worsening renal function accompanied by decongestion is not associated with worse outcomes, whereas patients with worsening renal function and residual congestion have a particularly poor prognosis (26, 27). However, few studies have examined the interesting relationship between changes in NT-proBNP and kidney dysfunction in patients with CHF. In this study, 41.5% of elderly patients died within 90 days of AKI onset. The more severe stages of AKI were shown to predict mortality and renal outcomes in elderly patients with CHF–independent of NT-proBNP levels. The decline of renal function should be avoided in patients with CHF.

In this study, 18% of survivors did not fully recover from AKI. Compared with non-elderly patients, impaired recovery of kidney function after AKI is more common in elderly patients (28). Non-recovery from AKI in elderly patients leaves this population at a higher risk of long-term morbidity and mortality (29). Therefore, avoiding AKI episodes and facilitating prompt prophylactic strategies in the elderly is vital to improve prognosis. A decreased change in NT-proBNP during AKI was shown to be a risk factor for the non-recovery of renal function–an effect that was most obvious in patients with low baseline NT-proBNP levels. In addition to HF, volume depletion (as indicated by decreased serum NT-proBNP levels) is another important cause of pre-renal AKI in the elderly. Fluid removal has been observed to increase the risk of worsening renal function in patients with acute HF (30). Elderly patients are more prone to dehydration, owing to impaired renal concentrating ability, diuretic use, and restricted ability to access fluids (6). According to our study, a decrease in NT-proBNP was found to prevent severe AKI in patients with CHF. However, a decrease in NT-proBNP and the use of loop diuretics predicted a higher risk of non-recovery of renal function in patients with AKI in CHF–especially in patients with low baseline NT-proBNP levels. Thus, excessive fluid removal may result in the non-recovery of the kidneys. Continuous monitoring of NT-proBNP is recommended in elderly patients with CHF. Avoiding excessive changes in NT-proBNP may be protective for the survival and renal injury prognosis.

There were several limitations of our study. This was a retrospective study in a single center that was restricted to a former member of the armed forces. Identified or unidentified confounders may have influenced the result. We lacked adequate data on the history of CHF (detailed echocardiogram indicators and vascular calcification) and the etiology of HF was difficult to precisely determine. We also lacked detailed data at AKI onset. In addition to BNP and extremity edema, lung congestion, liver congestion, and other indicators of volume and congestion status were unavailable and not included in our analyses. The causes of AKI and mortality in elderly patients with CHF are diverse and may have influenced the result. Subgroup analysis based on different causes is further required.

In conclusion, a decreased change in NT-proBNP was associated with a lower risk of more severe AKI in elderly patients with CHF. Elevated levels of baseline NT-proBNP and increased change in NT-proBNP accompanying the episode of AKI predicted a higher 90-day mortality in elderly patients with a CHF history. However, an excessive decrease in NT-proBNP is a risk factor for the non-recovery of renal function following AKI–especially in elderly CHF patients with low baseline NT-proBNP levels. Continuous monitoring of NT-proBNP is recommended in elderly patients with CHF and avoiding excessive changes in NT-proBNP may be protective for survival and renal injury prognosis.

## Data availability statement

The raw data supporting the conclusions of this article will be made available by the authors, without undue reservation.

## Ethics statement

The studies involving human participants were reviewed and approved by Ethics Committee of Chinese People’s Liberation Army General Hospital. Written informed consent for participation was not required for this study in accordance with the national legislation and the institutional requirements.

## Author contributions

JH and XZ contributed to the data analysis and drafting the manuscript. ZW contributed to the data collection and data analysis. YL, YZ, JZ, XW, HC, GY, and QM contributed to the data collection of the study. QC and QA contributed to the design of the study and data analysis. All authors reviewed the manuscript.
