# Supplementary material for: Association of serum N-terminal pro-brain natriuretic peptide levels with survival and renal outcomes among elderly patients with acute kidney injury in chronic heart failure
Source: Front Cardiovasc Med. 2023 Feb 3;10:1104787. doi: 10.3389/fcvm.2023.1104787 (PMC9935602; doi:10.3389/fcvm.2023.1104787)
Supplement: Supplementary file 1 [file Data_Sheet_1.docx]

**SUPPLEMENTAL MATERIAL**

**Table S1. Univariable and multivariable logistic regression analysis for more severe AKI (stage 2 and 3) by NT-proBNP levels.**

|  | Crude OR (95% CI) | Crude P value | Adjusted^a^ OR (95% CI) | Adjusted^a^ P value |
| --- | --- | --- | --- | --- |
| NT-proBNP _at baseline_ | | | | |
| ln | 0.87(0.77, 0.98) | 0.023 | 0.78(0.68-0.89) | < 0.001 |
| Q1 | Reference | - | Reference | - |
| Q2 | 0.75(0.47, 1.18) | 0.206 | 0.67(0.42, 1.08) | 0.101 |
| Q3 | 0.81(0.52, 1.27) | 0.364 | 0.63 (0.39, 1.02) | 0.062 |
| Q4 | 0.53(0.33, 0.87) | 0.012 | 0.38(0.22, 0.65) | 0.001 |
| Change in NT-proBNP | | | | |
| Ratio of NT-proBNP _at AKI_ to NT-proBNP _at baseline_ | 1.03 (1.02, 1.05) | <0.001 | 1.03 (1.01, 1.05) | 0.001 |
| Decreased change in NT-proBNP^b^ | 0.41(0.26,0.66) | <0.001 | 0.56(0.34-0.91) | 0.020 |

^a^ With adjustment for loop diuretics, ACEI/ARB, infection, fasting glucose, potassium, phosphorus, hemoglobin, systolic blood pressure and diastolic blood pressure.

^b^ Ratio of NT-proBNP _at AKI_/NT-proBNP _at baseline_ < 1 compared to ratio of NT-proBNP _at AKI_/ NT-proBNP _at baseline_ ≥ 1

**Supplemental Table S2. Full univariable and multivariable logistic regression analysis for more severe AKI (stage 2 and 3)**

|  | Univariate | | Multivariate | |
| --- | --- | --- | --- | --- |
|  | OR (95% CI) | p-value | OR (95% CI) | p-value |
| lnNT-proBNP _at baseline_ (pg/mL) | **0.87(0.76-0.98)** | **0.023** | **0.78(0.68-0.89)** | **< 0.001** |
| Decreased change in NT-proBNP ^c^ | **0.41(0.26-0.66)** | **< 0.001** | **0.56(0.34-0.91)** | **0.020** |
| Age (years) | 0.98(0.95-1.01) | 0.128 |  |  |
| Male | 1.10(0.64-1.89) | 0.719 | **0.51 (0.33-0.78)** | **0.002** |
| Baseline eGFR, (mL/min/1.73 m^2^) | 1.01(1.00-1.01) | 0.081 |  |  |
| 40%< LVEF < 50% | 1.00(0.67-1.50) | 0.983 |  |  |
| Loop diuretics | **1.88(1.34-2.65)** | **< 0.001** | **1.79(1.24-2.60)** | **0.002** |
| ACEI or ARB | **0.60(0.39-0.92)** | **0.019** | 0.72(0.46-1.12) | 0.143 |
| Aldosterone antagonist | 0.79(0.54-1.17) | 0.243 |  |  |
| Beta-blockers | 0.81(0.51-1.30) | 0.392 |  |  |
| Hypertension | 0.84(0.57-1.24) | 0.383 |  |  |
| Diabetes mellitus | 0.87(0.62-1.21) | 0.400 |  |  |
| Hyperlipidemia | 0.94(0.67-1.32) | 0.727 |  |  |
| Coronary heart disease | 0.83 (0.57-1.31) | 0.429 |  |  |
| Prior myocardial infarction | 1.02(0.70-1.47) | 0.927 |  |  |
| Atrial fibrillation | 1.05(0.73-1.51) | 0.796 |  |  |
| COPD | 0.99(0.70-1.40) | 0.951 |  |  |
| Malignant tumor | 1.19(0.85-1.67) | 0.315 |  |  |
| Proteinuria | 1.16(0.83-1.63) | 0.389 |  |  |
| Extremity edema | 1.04(0.74-1.47) | 0.811 |  |  |
| Infection* | **1.78(1.27-2.49)** | **0.001** | **1.48(1.04-2.12)** | **0.032** |
| Fasting glucose (mmol/L) | **1.05(1.02-1.09)** | **0.001** | **1.04(1.01-1.08)** | **0.015** |
| Potassium (mmol/L) | **1.49(1.21-1.84)** | **< 0.001** | 1.15(0.91-1.45) | 0.243 |
| Calcium (mmol/L) | 0.88(0.46-1.69) | 0.695 |  |  |
| Phosphorus (mmol/L) | **2.28(1.64-3.18)** | **< 0.001** | **2.14(1.50-3.05)** | **< 0.001** |
| Hemoglobin (g/dL) | **0.99(0.98-1.00)** | **0.004** | **0.99(0.98-1.00)** | **0.036** |
| cTnI (μg/L) | 1.01(1.00-1.03) | 0.158 |  |  |
| Systolic blood pressure (mmHg) | **0.99(0.98-0.99)** | **0.001** | 0.99(0.98-1.00) | 0.184 |
| Diastolic blood pressure (mmHg) | **0.98(0.97-1.00)** | **0.011** | 1.00(0.98-1.02) | 0.750 |

**Bolded values have p-value < 0.05.**

**Supplemental Table S3. Full univariable and multivariable Cox regression analysis for 90-day mortality.**

|  | Univariate | | Multivariate | |
| --- | --- | --- | --- | --- |
|  | HR (95% CI) | p-value | HR (95% CI) | p-value |
| lnNT-proBNP _at baseline_ (pg/mL) | **1.37(1.28-1.46)** | **< 0.001** | **1.27(1.17-1.38)** | **< 0.001** |
| Decreased change in NT-proBNP ^c^ | **0.55(0.44-0.70)** | **< 0.001** | **0.62(0.48-0.79)** | **< 0.001** |
| More severe AKI | **2.02(1.62-2.52)** | **< 0.001** | **1.52(1.20-1.93)** | **0.001** |
| Age (years) | 1.01(1.00-1.03) | 0.085 |  |  |
| Male | 1.17(0.87-1.57) | 0.297 |  |  |
| Baseline eGFR, (mL/min/1.73 m^2^) | 1.00(0.99-1.00) | 0.360 |  |  |
| 40%< LVEF < 50% | **2.27(1.88-2.74)** | **< 0.001** | **1.56(1.26-1.92)** | **< 0.001** |
| Loop diuretics | **1.85(1.54-2.22)** | **< 0.001** | 1.20(0.99-1.46) | 0.060 |
| ACEI or ARB | **0.38(0.30-0.49)** | **< 0.001** | **0.46(0.35-0.60)** | **< 0.001** |
| Aldosterone antagonist | 0.92(0.75-1.13) | 0.412 |  |  |
| Beta-blockers | 0.82(0.64-1.05) | 0.112 |  |  |
| Hypertension | **0.72(0.59-0.88)** | **0.001** | 1.00(0.80-1.24) | 0.968 |
| Diabetes mellitus | 0.86(0.72-1.03) | 0.093 |  |  |
| Hyperlipidemia | **0.76(0.63-0.91)** | **0.003** | 0.84(0.70-1.01) | 0.063 |
| Coronary heart disease | **0.75(0.60-0.94)** | **0.013** | 0.87(0.73-1.02) | 0.096 |
| Prior myocardial infarction | **1.47(1.22-1.78)** | **< 0.001** | 1.13(0.91-1.40) | 0.269 |
| Atrial fibrillation | **1.25(1.03-1.51)** | **0.022** | 1.11(0.91-1.35) | 0.312 |
| COPD | **1.20(1.00-1.44)** | **0.048** | 1.17(0.97-1.42) | 0.108 |
| Malignant tumor | **1.72(1.44-2.05)** | **< 0.001** | **1.47(1.22-1.78)** | **< 0.001** |
| Proteinuria | 1.20(1.00-1.44) | 0.052 |  |  |
| Extremity edema | **1.62(1.35-1.94)** | **< 0.001** | 1.05(0.86-1.28) | 0.628 |
| Infection* | **2.04(1.70-2.44)** | **< 0.001** | **1.45(1.20-1.75)** | **< 0.001** |
| Fasting glucose (mmol/L) | **1.05(1.03-1.06)** | **< 0.001** | **1.04(1.02-1.06)** | **< 0.001** |
| Potassium (mmol/L) | **1.31(1.17-1.48)** | **< 0.001** | 1.09(0.96-1.23) | 0.187 |
| Calcium (mmol/L) | **0.50(0.34-0.72)** | **< 0.001** | 1.06(0.73-1.53) | 0.771 |
| Phosphorus (mmol/L) | **1.86(1.56-2.23)** | **< 0.001** | **1.28(1.07-1.53)** | **< 0.001** |
| Hemoglobin (g/dL) | **0.98(0.97-0.98)** | **< 0.001** | **0.98(0.98-0.99)** | **< 0.001** |
| cTnI (μg/L) | **1.02(1.01-1.02)** | **< 0.001** | **1.01(1.00-1.02)** | **0.003** |
| Systolic blood pressure (mmHg) | **0.98(0.98-0.98)** | **< 0.001** | **0.99(0.98-1.00)** | **< 0.001** |
| Diastolic blood pressure (mmHg) | **0.97(0.96-0.98)** | **< 0.001** | 1.00(0.99-1.01) | 0.547 |

**Bolded values have p-value < 0.05.**

**Supplemental Table S4. Full univariable and multivariable logistic regression analysis for 90-day renal non-recovery**

|  | Univariate | | Multivariate | |
| --- | --- | --- | --- | --- |
|  | OR (95% CI) | p-value | OR (95% CI) | p-value |
| lnNT-proBNP _at baseline_ (pg/mL) | 1.04(0.89-1.21) | 0.610 | 1.01(0.84-1.21) | 0.921 |
| Decreased change in NT-proBNP ^c^ | **1.55(1.03-2.33)** | **0.034** | **1.59(1.02-2.48)** | **0.041** |
| More severe AKI | **1.88(1.04-3.43)** | **0.038** | **2.11(1.09-4.10)** | **0.026** |
| Age (years) | 1.00(0.96-1.03) | 0.828 |  |  |
| Male | 0.99(0.55-1.81) | 0.984 |  |  |
| Baseline eGFR, (mL/min/1.73 m^2^) | **1.02(1.01-1.03)** | **0.001** | **1.02(1.01-1.03)** | **< 0.001** |
| 40%< LVEF < 50% | 1.16(0.67-2.03) | 0.595 |  |  |
| Loop diuretics | **1.75(1.14-2.69)** | **0.010** | **2.45(1.49-4.02)** | **< 0.001** |
| ACEI or ARB | 1.27(0.85-1.90) | 0.252 |  |  |
| Aldosterone antagonist | 0.77(0.49-1.21) | 0.262 |  |  |
| Beta-blockers | 0.87(0.52-1.48) | 0.611 |  |  |
| Hypertension | 0.89(0.55-1.44) | 0.636 |  |  |
| Diabetes mellitus | 1.03(0.70-1.53) | 0.872 |  |  |
| Hyperlipidemia | 1.25(0.84-1.85) | 0.270 |  |  |
| Coronary heart disease | 0.82(0.48-1.40) | 0.468 |  |  |
| Prior myocardial infarction | 1.02(0.65-1.60) | 0.942 |  |  |
| Atrial fibrillation | **1.53(1.01-2.34)** | **0.047** | 1.45(0.91-2.30) | 0.117 |
| COPD | 0.93(0.62-1.41) | 0.741 |  |  |
| Malignant tumor | **1.61(1.07-2.41)** | **0.023** | 1.41(0.91-2.19) | 0.124 |
| Proteinuria | **1.84(1.22-2.76)** | **0.003** | **1.82(1.17-2.86)** | **0.009** |
| Extremity edema | 0.97(0.63-1.49) | 0.877 |  |  |
| Infection* | 0.79(0.52-1.20) | 0.267 |  |  |
| Fasting glucose (mmol/L) | **0.94(0.88-1.00)** | **0.037** | **0.92(0.87-0.98)** | **0.009** |
| Potassium (mmol/L) | 1.00(0.75-1.34) | 0.996 |  |  |
| Calcium (mmol/L) | **2.63(1.13-6.11)** | **0.025** | 2.45(0.94-6.40) | 0.067 |
| Phosphorus (mmol/L) | 1.07(0.63-1.82) | 0.799 |  |  |
| Hemoglobin (g/dL) | **0.99(0.98-1.00)** | **0.018** | 0.99(0.98-1.00) | 0.095 |
| cTnI (μg/L) | 0.96(0.88-1.05) | 0.347 |  |  |
| Systolic blood pressure (mmHg) | 1.00(0.99-1.01) | 0.649 |  |  |
| Diastolic blood pressure (mmHg) | 0.99(0.98-1.01) | 0.523 |  |  |

**Bolded values have p-value < 0.05.**
